# Partial Oral Versus Intravenous Antibiotic Therapy for Endocarditis With Management by a Multidisciplinary Team: A Retrospective Cohort Study

**DOI:** 10.1093/ofid/ofaf625

**Published:** 2025-10-28

**Authors:** Sami El-Dalati, Bennett Collis, Takaaki Kobayashi, Evan Hall, Talal Alnabelsi, Chloe Cao, Meredith Johnson, John Gurley, Luke Strnad, Corey Adams, Victoria Weaver, Hassan Reda, Michael Sekela, Tessa London, Kara Kennedy, Armaghan-E Rehman Mansoor, David Olafsson, Grant Laugherty, Alyssa Tremblay, Angella Linder, Deborah Gill, Nicholas J Van Sickels, Alexander Pomakov, William Harris, Bobbi Jo Stoner

**Affiliations:** Division of Infectious Diseases, Department of Internal Medicine, University of Kentucky Medical Center, Lexington, Kentucky, USA; University of Kentucky College of Medicine, Lexington, Kentucky, USA; Division of Infectious Diseases, Department of Internal Medicine, University of Kentucky Medical Center, Lexington, Kentucky, USA; University of Kentucky College of Medicine, Lexington, Kentucky, USA; Division of Cardiology, Department of Internal Medicine, University of Kentucky Medical Center, Lexington, Kentucky, USA; Department of Internal Medicine, HCA Healthcare/University of South Florida Morsani College of Medicine Graduate Medical Education/HCA Florida Trinity, Tampa, Florida, USA; Department of Surgery, University of Kentucky Medical Center, Lexington, Kentucky, USA; Division of Cardiology, Department of Internal Medicine, University of Kentucky Medical Center, Lexington, Kentucky, USA; Division of Infectious Diseases, Department of Internal Medicine, Oregon Health and Science University, Portland, Oregon, USA; Department of Cardiac Sciences, Libin Cardiovascular Institute, University of Calgary, Calgary, Alberta, Canada; Division of Infectious Diseases, Department of Medicine, University of British Columbia, Vancouver, British Columbia, Canada; Division of Infectious Diseases, Department of Medicine, University of British Columbia, Vancouver, British Columbia, Canada; Division of Cardiac Surgery, University of Kentucky Medical Center, Lexington, Kentucky, USA; Division of Infectious Diseases, Department of Medicine, University of British Columbia, Vancouver, British Columbia, Canada; Department of Neurology, University of Kentucky, Lexington, Kentucky, USA; Division of Infectious Diseases, Department of Internal Medicine, University of Kentucky Medical Center, Lexington, Kentucky, USA; Division of Infectious Diseases, Department of Internal Medicine, University of Kentucky Medical Center, Lexington, Kentucky, USA; Division of Infectious Diseases, Department of Internal Medicine, University of Kentucky Medical Center, Lexington, Kentucky, USA; Division of Infectious Diseases, Department of Internal Medicine, University of Kentucky Medical Center, Lexington, Kentucky, USA; Division of Infectious Diseases, Department of Internal Medicine, University of Kentucky Medical Center, Lexington, Kentucky, USA; Division of Infectious Diseases, Department of Internal Medicine, University of Kentucky Medical Center, Lexington, Kentucky, USA; Division of Infectious Diseases, Department of Internal Medicine, University of Kentucky Medical Center, Lexington, Kentucky, USA; Division of Infectious Diseases, Department of Internal Medicine, University of Kentucky Medical Center, Lexington, Kentucky, USA; University of Kentucky College of Pharmacy, Lexington, Kentucky, USA; University of Kentucky College of Pharmacy, Lexington, Kentucky, USA

**Keywords:** endocarditis, multidisciplinary teams, oral antibiotic treatment, patients who inject drugs

## Abstract

**Background:**

Despite trial data supporting oral stepdown therapy for infective endocarditis (IE), its use remains limited, especially in North America. We evaluated outcomes of patients with IE managed by a multidisciplinary team and treated with either intravenous (IV) or partial oral antibiotics.

**Methods:**

This was a single-center retrospective study of patients with definite IE identified from an institutional registry between 7 September 2021 and 1 March 2025. Clinical and outcomes data were analyzed using multivariable logistic regression.

**Results:**

Of 236 patients, 143 received IV therapy alone and 93 were transitioned to partial oral therapy. Baseline characteristics were similar, though valve surgery was more frequent in the oral group (40.9% vs 28.0%; *P* = .04). There were no significant differences in 90-day relapsed infection (0.7% vs 2.2%; *P* = .32), 90-day all-cause mortality (2.8% vs 6.5%; *P* = .17), or the composite of both outcomes (3.5% vs 8.6%; *P* = .09). There was no difference in relapsed infection or all-cause mortality at 90 days for patients with methicillin-resistant *Staphylococcus aureus* transitioned to oral therapy. In multivariable analysis, oral therapy was not associated with increased 90-day mortality (odds ratio [OR], 1.72 [95% confidence interval {CI} .41–7.24]; *P* = .46). Independent predictors of mortality included older age (OR, 1.06 per year [95% CI, 1.00–1.13]; *P* < .001), acute heart failure (OR, 18.61), and discharge before medically advised (OR, 8.60).

**Conclusions:**

In selected patients managed by a multidisciplinary team, partial oral therapy for IE appears to be safe and effective, with outcomes comparable to exclusive IV treatment, consistent with European guidelines.

Infective endocarditis (IE) is associated with significant morbidity and mortality as well as cost to the United States (US) healthcare system [1, 2]. In recent years, literature has demonstrated that partial oral antimicrobial therapy is noninferior to treatment regimens delivered entirely intravenously and no published study has found that intravenous (IV) antibiotics are superior to oral in treatment of IE [3–6]. While European IE guidelines have incorporated recommendations for oral antibiotics in certain cases, the American Heart Association (AHA) IE guideline has not been formally updated since 2015 [7, 8]. The studies that have emphasized the role of oral antibiotic therapy had very rigorous screening and follow-up requirements as well as relatively small numbers of persons who inject drugs (PWIDs). For example, Iversen et al performed a randomized controlled trial requiring patients assigned to oral treatment to follow up on an outpatient basis 2–3 times per week, and all patients underwent transesophageal echocardiography (TEE) 1–3 days prior to oral switch to rule out progression of disease. The investigators also excluded patients with right-sided IE. Their study only included 5 PWIDs out of 400 participants and was notably conducted in Denmark, which has a substantially different healthcare system and network of social services compared to the US [3]. A follow-up study of 562 patients by the same group reported similar outcomes but again only included left-sided IE and just 10 PWIDs [6]. Freling et al performed a retrospective cohort study in the US after implementing an expected practice for the use of oral antibiotics and demonstrated similar outcomes for patients treated with partial oral versus IV therapy, but without the use of a multidisciplinary endocarditis team or details about how patients were selected for each treatment strategy [4]. Additionally, there were only 46 patients in the oral arm of that retrospective study, nearly 40% had possible endocarditis, only 37% had a history of injection drug use (IDU), and <10% had prosthetic valves. Consequently, questions remain about the applicability of existing data to the PWID population and how to realistically implement oral therapy as part of clinical practice [9]. We previously reported on 32 patients with endocarditis treated with oral antimicrobials by a multidisciplinary endocarditis team over a 1-year period [10]. However, that study did not have an IV control group and was limited by the relatively low number of patients. Here we report our approximately 4-year experience using a multidisciplinary endocarditis team to select patients for partial oral antibiotic therapy and compare the treatment outcomes to those of patients receiving IV treatment only.

## METHODS

Institutional review board (IRB) approval was obtained from the University of Kentucky. Patient consent was not required by the IRB. In September 2021, University of Kentucky Healthcare (UK) created a multidisciplinary endocarditis team (MDET) and cardiovascular infectious diseases consult service (CVIDCS). The structure and function of the MDET and CVIDCS have been explained in detail previously [10–14]. All patients admitted to UK with IE are seen by the CVIDCS and discussed at the weekly MDET conference. All patients with active substance use disorder or a history of IDU are offered addiction medicine consultation. Decisions regarding antibiotic route and duration, percutaneous mechanical aspiration, and valve surgery are made at the MDET weekly meeting. The approach to offering patients oral antibiotics was described in our previous publication and included a detailed protocol, derived from Iversen et al and since adopted by the European Society of Cardiology (ESC) [3, 7, 10]. In general, once a patient with IE caused by methicillin-susceptible *Staphylococcus aureus* (MSSA), coagulase-negative staphylococci (CoNS), *Enterococcus faecalis*, or streptococci was deemed stable for discharge by the MDET and had received at least 10 days of IV antibiotics from blood culture clearance or 7 days from valve surgery, they were offered the option of completing treatment with oral antibiotics or continuing with IV therapy either in the hospital, in a skilled nursing facility, or via outpatient parenteral antibiotic therapy (OPAT). TEE was not required prior to oral switch. Durations of therapy were based on guideline recommendations from the AHA and ESC [7, 8].

Patients with the aforementioned organisms who were switched to oral therapy were primarily treated with 2 different oral agents with different mechanisms of action [3, 6, 7]. In contrast, patients with methicillin-resistant *Staphylococcus aureus* (MRSA) were offered monotherapy with oral linezolid, as supported by the clinical experience reported by Freling et al [4]. There was limited data to support the use of 2 oral agents for treatment of MRSA endocarditis.

Some patients, due to the acuity of their illness, were not clinically stable for discharge until well after they completed their IE antimicrobial treatment. Patients referred to acute or subacute rehabilitation facilities based on their physical and occupational therapy needs were offered oral antimicrobials if their anticipated rehabilitation stay was shorter than the expected duration of antimicrobial therapy.

If a patient preferred to receive a full course of IV treatment, they were then evaluated by the OPAT team. The OPAT group at UK predates the existence of the MDET and CVIDCS and does not routinely attend MDET meetings. Once a referral is made to the OPAT team, the patient is then seen by an OPAT nurse navigator who performs an evaluation of the patient’s social situation and ability to perform infusions either independently or with the assistance of a friend/family member. Substance use is not considered an absolute contraindication to enrolling in OPAT, but depending on the infectious diseases provider it may lead to the patient being declined. Based on these factors, the OPAT team makes a final assessment regarding whether the patient can discharge home to receive IV antimicrobials. Patients discharged to lower-acuity medical facilities are still enrolled in OPAT for the purposes of laboratory monitoring but do not undergo a nurse navigator evaluation. Using this shared decision-making approach, the CVIDCS and patients collaborated on whether to complete antibiotic therapy with oral or IV regimens [10].

Individuals who left the hospital before medically advised (BMA), regardless of the organism, were also offered oral antimicrobial treatment. Whenever possible, bedside medication delivery from the hospital's outpatient pharmacy was performed. In some instances, patients would depart their rooms before their medication could be delivered. In those cases, their oral antibiotics were sent to their preferred ambulatory pharmacy. For individuals who discharged BMA, if the investigators could not confirm they picked up their oral antibiotic prescription or if they did not present for follow-up, they were included in the IV arm and their total duration of therapy was calculated by the number of days of IV treatment they received [10].

Patients were scheduled for infectious diseases follow-up within 2 weeks of discharge. At these appointments, laboratory monitoring with complete blood counts and comprehensive metabolic panels was performed. Blood cultures were obtained if patients reported symptoms concerning for recurrent bloodstream infection [10].

### Patient Identification

Beginning in September 2021, a registry was created in the hospital's electronic medical record containing all patients presented at the weekly MDET conference. IRB approval was obtained from the University of Kentucky to establish a database to retrospectively collect each patient's demographic, comorbidities, diagnostics, treatments, and outcomes data. For patients who did not follow-up in clinic, data for mortality and reinfections were collected based on emergency department visits and admissions to either the authors' institution or other hospitals in the region. Follow-up data at 90 days were available for all patients.

**Figure 1.**
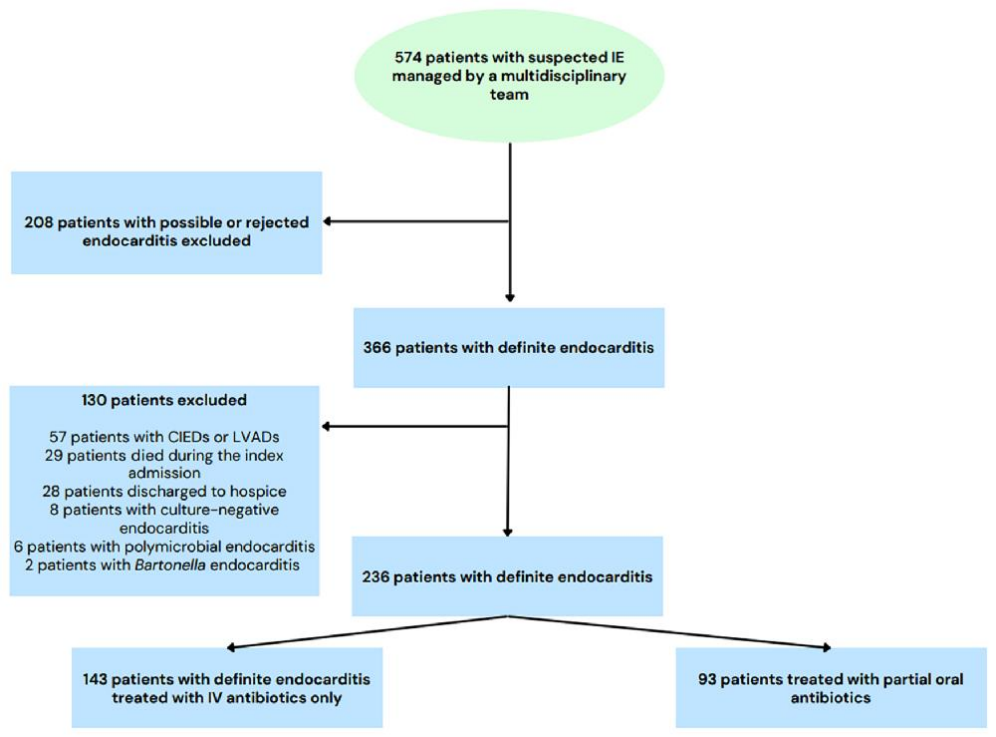
Study enrollment flowchart. Abbreviations: CIED, cardiac implantable electronic device; IE, infective endocarditis; IV, intravenous; LVAD, left ventricular assist device.

Patients were included in this study if they met the modified Duke criteria for definite IE caused by MRSA, MSSA, CoNS, *E faecalis*, streptococci, *Candida*, or gram-negative bacteria [15]. Patients were excluded if they had a cardiac implantable electronic device or left ventricular assist device, died during the index hospitalization or were discharged to hospice, or had culture-negative or polymicrobial endocarditis (Figure 1). Patients who received any duration of oral therapy for primary treatment of IE were included in the *per os* (PO) arm. The primary outcomes included 30-day and 90-day all-cause mortality as well as rates of relapsed infection at 30 days and 90 days. An additional primary outcome was a composite outcome of all-cause mortality and relapsed infection at 90 days. Secondary outcomes included 30- and 90-day readmission rates.

The reporting of this study conformed to the Strengthening the Reporting of Observational Studies in Epidemiology (STROBE) statement [16].

### Definition of Terms

Active IDU was defined as injection substance use within 30 days of the index hospitalization. Blood culture clearance was defined by the presence of 2 consecutive negative blood cultures obtained on separate calendar days. The start date for treatment duration was defined from the date of blood culture clearance and durations of therapy were based on the recommendations from the CVIDCS and MDET. Relapsed infection was defined by the presence of a new positive blood culture with the same organism from the index hospitalization after previously documenting negative cultures. Duration of oral antibiotic treatment was confirmed with patients at follow-up. For those who did not follow-up, duration was assumed, based on the number of days they received in the hospital plus the length of the oral antibiotic prescription. Pickup of the prescription was confirmed with the hospital's pharmacy. Consolidative antibiotic therapy was defined as oral antibiotic therapy provided after completion of an initial course of treatment for IE for patients with *Staphylococcus aureus* vertebral osteomyelitis with the aim of preventing relapsed infection. Suppressive antibiotic therapy was defined as oral antibiotic therapy provided after completion of an initial course of treatment for IE for patients with prosthetic valve IE with the aim of preventing relapsed or progressive infection. Acute renal failure was defined as patients who were initiated on renal replacement therapy during the index hospitalization.

### Analysis

To assess whether an oral switch for primary IE treatment is associated with adverse clinical outcomes, we conducted a logistic regression analysis. Given the low number of deaths within 30 days, 90-day mortality was used as the outcome. We first performed univariable analyses using available variables and included those with a *P* value <.2 in the multivariable logistic regression model. Statistical significance was defined as *P* < .05. All analyses were conducted using Stata Statistical Software: Release 16 (StataCorp LLC, College Station, Texas).

## RESULTS

### Demographics

A total of 236 patients with definite IE were included between 7 September 2021 and 1 March 2025; 143 (60.6%) received only IV antibiotics and 93 (39.4%) were transitioned to PO therapy (Figure 1). Median age was 43 years (interquartile range [IQR], 33.5–54.5 years) in the IV group and 39 years (IQR, 33–50 years) in the PO group (*P* = .10; Table 1). Male sex was 59.4% (IV) versus 54.8% (PO) (*P* = .57). The only significant baseline difference was chronic dialysis, which was more common in the IV group (6.3% vs 0%; *P* = .01). Recent IDU was reported in 58.0% (IV) versus 57.0% (PO) (*P* = .88), and prior IE in 30.1% versus 31.2% (*P* = .86). Prosthetic valves were present in 14.0% (IV) versus 17.2% (PO) (*P* = .51). Tricuspid valve involvement was most frequent (44.8% IV vs 47.4% PO; *P* = .70), followed by mitral valves (37.1% vs 30.1%; *P* = .28) and aortic valves (32.2% vs 31.2%; *P* = .87). Multivalvular IE was present in 15.4% (IV) versus 12.9% (PO) (*P* = .59). Intensive care unit admission rates were 55.2% (IV) versus 64.5% (PO) (*P* = .16), and neurologic complications occurred in 37.1% versus 31.2% (*P* = .34).

**Table 1.** Demographic Information for Patients Treated Exclusively With Intravenous Antimicrobials and Those Transitioned to Oral Therapy

Variable	IV Only Therapy (n = 143)	IV With Oral Course Completion Therapy (n = 93)	*P* Value
Age, y, median (IQR)	43 (33.5–54.5)	39 (33–50)	.10
Sex			.57
Male	59.4 (85)	54.8 (51)	
Female	40.6 (58)	45.2 (42)	
Race/ethnicity			.34
White	91.6 (131)	96.8 (90)	
Black	4.9 (7)	1.1 (1)	
Hispanic	2.8 (4)	2.2 (2)	
Multiracial	0.7 (1)	0 (0)	
Recent injection drug use	58.0 (83)	57.0 (53)	.88
Hepatitis C viremia	35.7 (51)	28.0 (26)	.22
Type 2 diabetes mellitus	21.0 (30)	14.0 (13)	.17
Dental disease	39.9 (57)	46.2 (43)	.34
Chronic dialysis	6.3 (9)	0 (0)	.01
Previous admission for IE	30.1 (43)	31.2 (29)	.86
Prosthetic valve	14.0 (20)	17.2 (16)	.51
Outside hospital transfer	45.5 (65)	53.8 (50)	.21
ID consult	100 (143)	100 (93)	
Days from admission to ID consult, median (IQR)	1 (1–2)	1 (1–2)	.08
Addiction medicine consult	64.3 (92)	68.8 (64)	.48
Cardiothoracic surgery consult	82.5 (118)	83.9 (78)	.78
Days from admission to cardiothoracic surgery consult, median (IQR)	2 (1–4)	1.5 (0–2.8)	.10
Aortic valve vegetation	32.2 (46)	31.2 (29)	.87
Mitral valve vegetation	37.1 (53)	30.1 (28)	.28
Tricuspid valve vegetation	44.8 (64)	47.4 (44)	.70
Pulmonic valve vegetation	2.1 (3)	0 (0)	.16
Multivalvular vegetation	15.4 (22)	12.9 (12)	.59
Pitt bacteremia score, median (IQR)	0 (0–2)	0 (0–2)	.68
Acute heart failure secondary to IE	36.4 (52)	44.1 (41)	.24
Septic pulmonary emboli	46.2 (66)	44.1 (41)	.75
Ischemic stroke	21.0 (30)	20.4 (19)	.91
Intracranial hemorrhage	16.1 (23)	10.8 (10)	.25
Mycotic aneurysm	4.9 (7)	5.4 (5)	.86
Meningitis	2.1 (3)	0 (0)	.16
Epidural abscess	4.2 (6)	2.2 (2)	.41
Vertebral osteomyelitis	11.9 (17)	12.9 (12)	.82
ICU admission	55.2 (79)	64.5 (60)	.16
Days of ICU stay, median (IQR)	11 (4–15)	8 (4–13)	.06
Acute renal replacement therapy	6.3 (9)	4.3 (4)	.51
Vasopressor requirement	28.7 (41)	36.6 (34)	.20
Mechanical ventilation	42.0 (60)	49.5 (46)	.26
Percutaneous mechanical aspiration	3.5 (5)	5.4 (5)	.48
Valve surgery performed	28.0 (40)	40.9 (38)	.04

Data are presented as % (No.) unless otherwise indicated.

Abbreviations: ICU, intensive care unit; ID, infectious diseases; IE, infective endocarditis; IQR, interquartile range; IV, intravenous.

### Microbiology

MRSA was the most common pathogen in the IV group, isolated in 33.6% of patients compared to 25.8% (*P* = .20) in the oral arm (Table 2). MSSA was the most common pathogen in the PO group at 28%, although the distribution of MSSA cases was similar in the IV arm (29.4%; *P* = .82). There was a significantly higher percentage of patients with *E faecalis* IE treated with oral therapy (21.5% vs 8.4%; *P* = .004). There were similar rates of IE caused by streptococci and CoNS. More cases of non-Haemophilus, Aggregatibacter, Cardiobacterium, Eikenella, Kingella (HACEK) gram-negative IE were treated with IV antibiotics (7% vs 2.1%; *P* = .09) and there were low rates of HACEK and *Candida* IE in both cohorts. A detailed breakdown of pathogens is included in Supplementary Table 1.

**Table 2.** Microbiology of Patients Treated Exclusively With Intravenous Antimicrobials and Those Transitioned to Oral Therapy

Organism	IV Only Therapy (n = 143)	IV With Oral Course Completion Therapy (n = 93)	*P* Value
MRSA	33.6 (48)	25.8 (24)	.20
MSSA	29.4 (42)	28.0 (26)	.82
CoNS	3.5 (5)	3.2 (3)	.90
*Streptococcus* spp	15.4 (22)	18.3 (17)	.56
*Enterococcus* spp	8.4 (12)	21.5 (20)	.001
*Serratia* spp	5.6 (8)	1.1 (1)	.08
*Candida* spp	1.4 (2)	0 (0)	.25
*Pseudomonas* spp	1.4 (2)	1.1 (1)	.84
*Cardiobacterium hominis*	0 (0)	1.1 (1)	.21
*Aggregatibacter aphorphilus*	0.7 (1)	0 (0)	.42
*Rothia mucilaginosa*	0.7 (1)	0 (0)	.42

Data are presented as % (No.).

Abbreviations: CoNS, coagulase-negative staphylococci; IV, intravenous; MRSA, methicillin-resistant *Staphylococcus aureus*; MSSA, methicillin-susceptible *Staphylococcus aureus*.

### Antimicrobial Therapy

All patients who were transitioned to oral antimicrobials received an initial course of IV therapy, and the median duration of IV lead-in treatment was 18 days (IQR, 12–27 days; Table 3). The median duration of partial oral antibiotic therapy for treatment of IE was 14 days (IQR, 10–23 days). The median duration of total antimicrobial therapy was 41 days in both groups. Thirty-eight patients (26.4%) treated with only IV antimicrobials were transitioned to suppressive/consolidative oral treatment after completing an index course of therapy compared to 15 patients (16.1%) in the partial PO group (*P* = .06). The median duration of suppressive/consolidative therapy in the IV arm was 92 days (IQR, 73.3–122.3 days) compared to 81 days (IQR, 52.5–185 days) in the PO arm (*P* = .11). More patients who were transitioned to oral therapy underwent valve surgery during the index hospitalization (40.9% vs 28%; *P* = .04).

**Table 3.** Antimicrobial Duration for Patients Treated Exclusively With Intravenous Antimicrobials and Those Transitioned to Oral Therapy

Variable	IV Only Therapy (n = 143)	IV With Oral Course Completion Therapy (n = 93)	*P* Value
IV antimicrobial therapy	143 (100)	100 (93)	
Course completion oral antimicrobial therapy	NA	100 (93)	
Days of IV therapy prior to oral course completion, median (IQR)	NA	18 (12–27)	NA
Days of oral course completion, median (IQR)	NA	14 (10–23)	NA
Total days of antimicrobial therapy (IV vs IV with oral), median (IQR)	41 (27–41)	41 (29–42)	1.0
Consolidation oral antimicrobial therapy	26.6 (38)	16.1 (15)	.06
Days of oral consolidation antimicrobial therapy, median (IQR)	92 (73.3–122.3)	81 (52.5–185)	.11

Data are presented as % (No.) unless otherwise indicated.

Abbreviations: IQR, interquartile range; IV, intravenous; NA, not applicable.

Detailed breakdowns of antibiotic therapy for patients with MRSA, MSSA, CoNS, *E faecalis*, and streptococci are available in Supplementary Tables 2–5.

### Patient Outcomes

The median length of stay was 6 days shorter among patients treated with oral antibiotics (28 days vs 22 days; *P* = .001; Table 4). Approximately 15% of patients in both groups discharged BMA. There was no significant difference in 30-day (0.7% vs 1.1%; *P* = .75) and 90-day all-cause mortality (2.8% vs 6.5%; *P* = .17) between the IV and PO groups. Similarly, there was no significant difference in rates of relapsed infection at 30 days (0.7% vs 0%; *P* = .42) and 90 days (0.7% vs 2.2%; *P* = .32). There was no difference in the composite outcome of mortality or reinfection at 90 days between the IV and PO groups (3.5% vs 8.6%; *P* = .09). Readmission rates at 30 and 90 days were 30.1% and 36.4%, respectively, in the IV group and 22.6% and 32.3% in the PO arm. Eighty-percent of patients who transitioned to oral therapy followed up in the infectious diseases clinic compared to 70% of patients treated with IV (*P* = .10; Table 5).

**Table 4.** Clinical Outcomes for Patients Treated Exclusively With Intravenous Antimicrobials and Those Transitioned to Oral Therapy

Variable	IV Only Therapy (n = 143)	IV With Oral Course Completion Therapy (n = 93)	*P* Value
Total days of hospital admission, median (IQR)	28 (15–45)	22 (14–33)	.001
Discharge before medically advised	15.4 (22)	15.1 (14)	.95
30-d relapsed infection	0.7 (1)	0 (0)	.42
30-d readmission	30.1 (43)	22.6 (21)	.21
30-d all-cause mortality	0.7 (1)	1.1 (1)	.75
90-d relapsed infection	0.7 (1)	2.2 (2)	.32
90-d readmission	36.4 (52)	32.3 (30)	.52
90-d all-cause mortality	2.8 (4)	6.5 (6)	.17
Composite of 90-d all-cause mortality and relapsed infection	3.5 (5)	8.6 (8)	.09

Data are presented as % (No.) unless otherwise indicated.

Abbreviations: IQR, interquartile range; IV, intravenous.

**Table 5.** Follow-up Rates for Patients Treated Exclusively With Intravenous Antimicrobials and Those Transitioned to Oral Therapy

Variable	IV Only Therapy (n = 143)	IV With Oral Course Completion Therapy (n = 93)	*P* Value
Discharge with medication for opioid use disorder	44.1 (63)	54.8 (51)	.11
UK infectious diseases outpatient follow-up	69.9 (100)	79.6 (74)	.10
UK cardiothoracic surgery outpatient follow-up	56.6 (81)	64.5 (60)	.23
UK cardiology outpatient follow-up	30.1 (43)	30.1 (28)	1.0
No UK outpatient follow-up	22.4 (32)	16.1 (15)	.24

Data are presented as % (No.).

Abbreviations: IV, intravenous; UK, University of Kentucky.

Among the deaths at 90 days in the PO group, 3 patients died from complications of heart failure after they were deemed to not be candidates for valve surgery, 2 patients died of suspected opioid overdose, and 1 patient died who discharged BMA. Of the 2 relapsed infections at 90 days in the oral group, both occurred in patients with MRSA IE. One individual was prescribed an additional course of consolidative oral antibiotics for concurrent vertebral osteomyelitis and stopped this medication before recommended and was readmitted with relapsed MRSA bacteremia. The other patient completed a course of oral antibiotics but had ongoing IDU and was readmitted with MRSA bacteremia and tricuspid valve IE.

### Isolated Right-Sided IE

There were 87 patients with isolated right-sided IE, including 50 who received only IV antibiotics and 37 who transitioned to oral therapy (Supplementary Table 6). Both groups were similar with respect to their baseline demographics, although more patients in the IV only cohort had hepatitis C viremia (60% vs 35.1%; *P* = .02). Eighty-eight percent of patients in the IV arm had recent IDU compared to 83.8% in the oral group (*P* = .58). Detailed microbiologic data are available in Supplementary Table 7. Median length of stay and rates of patient-directed discharge and percutaneous mechanical aspiration were similar between both cohorts (Supplementary Table 8). There was no significant difference in 90-day mortality (0% vs 2.7%; *P* = .25) or 90-day relapsed infection (0% vs 5.4%; *P* = .10) between patients treated with IV and partial oral therapy, respectively. Both of the reinfection cases were previously described above. Infectious diseases follow-up occurred in 75.7% of patients who transitioned to oral antibiotics (Supplementary Table 9).

### Risk Factors Associated With 90-Day Mortality

In univariable analysis, age was associated with increased 90-day mortality (odds ratio [OR], 1.04 [95% confidence interval {CI}, 1.00–1.08]; *P* = .055) and reached significance in the multivariable model (OR, 1.06 [95% CI, 1.00–1.13]; *P* < .001) (Table 6). Acute heart failure due to IE was significantly associated with mortality in both univariable (OR, 15.21 [95% CI, 1.89–122.2]; *P* = .01) and multivariable analyses (OR, 18.61 [95% CI, 2.13–161.89]; *P* = .042). Leaving the hospital BMA was independently associated with increased mortality in the multivariable model (OR, 8.60 [95% CI, 1.34–55.15]; *P* = .02). Oral therapy was not significantly associated with 90-day mortality in either univariable (OR, 2.40 [95% CI, .66–8.7]; *P* = .19) or multivariable analysis (OR, 1.72 [95% CI, .41–7.24]; *P* = .46).

**Table 6.** Univariable and Multivariable Analyses Using 90-Day Mortality as the Outcome

Variable	Univariable Analysis			Multivariable Analysis		
	OR	(95% CI)	*P* Value	OR	(95% CI)	*P* Value
Age	1.04	(1.00–1.08)	.055	1.06	(1.00–1.13)	**<**.**001**
Sex						
Female	reference	NA	NA	…	…	
Male	1.11	(.30–4.03)	.88	…	…	
Race/ethnicity						
White	reference	NA	NA	…	…	
Black	NA^a^	NA	NA	…	…	
Hispanic	3.37	(.37–30.33)	.98	…	…	
Multiracial	NA^a^	NA	NA	…	…	
Recent injection drug use	0.73	(.20–2.58)	.62	…	…	
Vegetation involving >2 valves compared to involvement of a single valve	0.65	(.08–5.3)	.69	…	…	
MRSA	NA^a^	NA	NA	…	…	
Hepatitis C viremia	NA^a^	NA	NA	…	…	
Type 2 diabetes mellitus	0.49	(.06–3.95)	.50	…	…	
Dental disease	2.11	(.58–7.67)	.26	…	…	
Chronic dialysis	3.03	(.34–26.87)	.32	…	…	
Previous admission for IE	0.98	(.25–3.89)	.97	…	…	
Prosthetic valve	0.61	(.07–4.94)	.64	…	…	
Aortic valve vegetation	…	…		…	…	
Mitral valve vegetation	…	…		…	…	
Tricuspid valve vegetation	…	…		…	…	
Pulmonic valve vegetation	…	…		…	…	
Multivalvular vegetation	…	…		…	…	
Pitt bacteremia score	1.03	(.73–1.45)	.89	…	…	
Acute heart failure secondary to IE	15.21	(1.89–122.2)	.01	18.61	(2.13–161.89)	.**042**
Septic pulmonary emboli	0.29	(.06–1.39)	.12	0.40	(.06–2.89)	.36
Ischemic stroke	0.95	(.20–4.6)	.95	…	…	
Intracranial hemorrhage	0.67	(.08–5.50)	.71	…	…	
Mycotic aneurysm	NA^a^	NA	NA	…	…	
Meningitis	NA^a^	NA	NA	…	…	
Epidural abscess	NA^a^	NA	NA	…	…	
Vertebral osteomyelitis	1.84	(.37–9.1)	.45	…	…	
ICU admission	1.05	(.29–3.82)	.94	…	…	
Acute renal replacement therapy	1.98	(.23–16.95)	.53	…	…	
Vasopressor requirement	1.46	(.40–5.32)	.57	…	…	
Mechanical ventilation	0.81	(.22–2.95)	.75	…	…	
Percutaneous mechanical aspiration	NA^a^	NA	NA	…	…	
Consolidative/suppressive therapy	NA^a^	NA	NA	…	…	
Valve surgery performed	0.86	(.22–3.43)	.83	…	…	
Patient discharged BMA	2.51	(.62–10.19)	.19	8.60	(1.34–55.15)	.**02**
Oral switch	2.40	(.66–8.7)	.19	1.72	(.41–7.24)	.46

Values in bold correspond to *p*-vales <.05.

Abbreviations: BMA, before medically advised; CI, confidence interval; ICU, intensive care unit; IE, infective endocarditis; MRSA, methicillin-resistant *Staphylococcus aureus*; NA, not applicable; OR, odds ratio.

^a^These variables were excluded from univariate and multivariable analyses because the outcome occurred in only 1 group, resulting in separation and unstable estimates.

## DISCUSSION

In this single-center, retrospective cohort study of 236 patients with IE managed by a multidisciplinary team, there was no significant difference in 90-day all-cause mortality, 90-day relapsed infection, or the composite of both outcomes between 143 patients treated exclusively with IV antibiotics and 93 patients transitioned to oral therapy. Treatment failure rates were low in both groups, including among patients with MRSA. In the multivariable logistic regression model, independent risk factors for 90-day mortality included older age, leaving the hospital BMA, and acute heart failure due to IE. Transition to oral therapy was not associated with an increased risk of 90-day mortality compared to completion of therapy with IV antibiotics.

Our study is notable in several respects. First, it represents one of the largest North American cohorts of patients with IE treated with oral antibiotics, incorporating a multidisciplinary approach to identify appropriate candidates for stepdown therapy. Unlike the Partial Oral Treatment of Endocarditis (POET) trial by Iversen et al, our study did not mandate TEE prior to oral transition and involved less intensive outpatient follow-up, yet still observed low rates of mortality and relapse [3]. Additionally, >50% of patients in both groups had a history of IDU, and >40% had tricuspid valve IE—2 populations largely excluded from the POET trial [3]. Outcomes were similar for patients with isolated right-sided IE compared to the entire study population and follow-up rates were >75% despite >80% of patients having a history of recent IDU.

Although 90-day mortality was numerically higher in the oral therapy group, the difference was not statistically significant. Most deaths were attributed to heart failure complications or opioid overdose rather than treatment failure. Logistic regression analysis, adjusted for potential confounders, confirmed that oral stepdown therapy was not associated with 90-day mortality. Notably, the only 2 relapsed infections in the oral group occurred in patients who either prematurely discontinued their prescribed antibiotic course or had ongoing IDU. While these cases met the study's criteria for relapse, it is unclear whether they represent true failures of oral therapy.

Age, acute heart failure, and patient-directed discharge were significantly associated with 90-day mortality, consistent with prior literature [17, 18]. Schranz et al, using a nationally representative database, reported that discharge against medical advice occurred in nearly one-quarter of patients hospitalized with opioid-related endocarditis and was strongly associated with early readmission [18]. Similarly, Mishra et al demonstrated that patients leaving against medical advice were more likely to experience non–central nervous system embolic events, recurrent bacteremia, and frequent rehospitalizations, indicating increased morbidity in this population [19]. Given that early oral switch approved by the MDET was not associated with increased mortality in our study, whereas oral transition in the context of BMA discharge was, oral switch decisions should not be based solely on patient preference. Careful evaluation and selection of candidates by a multidisciplinary team are essential to ensure safety and treatment success. Our approach to transitioning patients to partial oral therapy (developed in 2021) is very similar to the algorithm developed by the 2023 ESC endocarditis guideline, with less reliance on repeat TEE prior to switching treatment (Table 7) [7].

**Table 7.** Comparison of University of Kentucky Protocol for Transitioning to Partial Oral Therapy With 2023 European Society of Cardiology Guidelines

Criteria	University of Kentucky	ESC Guidelines
Organisms: MSSA Streptococci *Enterococcus faecalis* CoNS	Yes	Yes
Recommend 2-drug regimen	Yes	Yes
Duration of IV lead-in therapy 10 d from blood culture clearance 7 d from valve surgery	Yes	Yes
Clinically stable for discharge Responding to treatment Afebrile >48 h	Yes	Yes
Laboratory parameters WBC count <15 000/µL CRP <25% of maximum or <20 mg/L	No	Yes
BMI <40 kg/m^2^ and able to take oral medications	Yes	Yes
Repeat TEE prior to switch	No	Yes
Follow-up frequency	Within 2 wk of discharge	Not clearly stated

Abbreviations: BMI, body mass index; CoNS, coagulase-negative staphylococci; CRP, C-reactive protein; ESC, European Society of Cardiology; IV, intravenous; MSSA, methicillin-susceptible *Staphylococcus aureus*; TEE, transesophageal echocardiography; WBC, white blood cell.

Patients received a median duration of 18 days of IV therapy prior to oral stepdown and length of stay was 6 days shorter among patients receiving oral treatment. Although OPAT has been shown to decrease length of stay for many patients, it requires coordinating with home health agencies or infusions centers for weekly lab draws and intravascular access management, and is generally less available to PWIDs [20, 21]. Oral antibiotics, by contrast, are typically less expensive and more readily available, factors that likely contributed to the shorter hospital stay in the oral therapy group. There were also 9 patients on chronic dialysis in the IV group compared to none who received PO therapy. For many patients on chronic dialysis with gram-positive IE, IV vancomycin or cefazolin can be administered during their dialysis sessions via their dialysis catheter or fistula, thereby avoiding the need for additional central access. This may have contributed to the MDET's decision to treat these patients with IV therapy only. Additionally, many oral antibiotics require dose adjustments with end-stage renal disease, which can make adherence more challenging.

MRSA was the causative pathogen in 25% of patients who received oral antibiotics. There were no deaths in this group, and relapse rates were similar to those in patients with MRSA IE treated with IV antibiotics. All patients with MRSA who transitioned to oral therapy were treated with linezolid monotherapy rather than combination therapy and this approach was primarily used in patients who discharged BMA. The median IV lead-in duration for these patients was 24 days—6 days longer than the cohort median and 14 days longer than that required in the POET trial [3]. Dual therapy was not offered to these patients in large part due to the longer IV lead-in times and the lack of clear data to support this approach. These findings suggest that in a subset of clinically stable patients who have received a substantial course of IV therapy, transitioning to oral linezolid may be a safe and effective option for MRSA IE. However, the optimal duration of IV lead-in therapy remains to be determined. The results also raise questions about whether dual oral therapy is truly needed for all cases of IE and is an area for further study.

More patients with *E faecalis* IE were transitioned to oral therapy, largely due to challenges in coadministering IV ampicillin and ceftriaxone. This regimen involves a high sodium and fluid load, given multiple times daily, which in the authors' experience can lead to volume overload, particularly in patients with IE and valvular regurgitation. Additionally, the regimen is difficult to administer in the outpatient setting. As a result, the CVIDCS and MDET teams favored oral antibiotic treatment in this subgroup to mitigate these complications.

Significantly more (40.9% vs 28%) patients underwent valve surgery in the oral therapy arm. Previous studies have demonstrated that no more than 2 weeks of antibiotic therapy is required for patients who have received valve surgery for IE and do not have metastatic sites of infection [22–24]. Once the valvular vegetation is removed and surgical source control is achieved, stable postoperative patients are optimal candidates for oral antibiotic transitions, or shorter durations of therapy overall, particularly as this avoids the placement of central venous access, which can increase the risk for subsequent infection in patients with prosthetic valves [25]. While the presence or absence of surgical valve replacement was not explicitly a part of the MDET protocol for selecting patients for partial oral therapy, the presence of surgical source control and the desire to avoid central access were at times considered when evaluating for oral antibiotic treatment. The high rate of surgery in the oral group may also have helped to improve clinical outcomes in this cohort as valve replacement has been associated with improved intermediate-term mortality [26, 27].

Despite growing evidence supporting the efficacy of partial oral antibiotic therapy for IE in select patients, uptake remains limited. A 2025 survey, primarily of European infectious diseases physicians, found that approximately half never transition patients with IE to oral therapy [28]. An update to the 2015 AHA IE guidelines, or a focused update specifically addressing the expanding literature on oral antibiotic use, may be necessary to support broader adoption of this strategy [8].

### Limitations

This study is primarily limited by its single-center, retrospective design, which carries the inherent risk of selection bias. Although the 2 treatment groups were generally similar, they were neither matched nor randomized, and unmeasured confounding may be present. Patients who died or were discharged to hospice during the index hospitalization were excluded. While this may introduce selection bias, none of these patients were transitioned to oral therapy prior to death or hospice enrollment; all received IV therapy until that point. Additionally, several variables were excluded from the logistic regression analysis due to separation issues, as the outcome occurred only in 1 group (eg, MRSA, mycotic aneurysm, meningitis, epidural abscess, race, percutaneous mechanical aspiration, and consolidative/suppressive therapy). These variables were observed exclusively among survivors. Given that death occurred in only 10 of 236 patients (4.2%), a meaningful assessment of their impact on mortality was not possible. However, since all excluded variables occurred only in survivors, their removal likely had minimal impact on the observed association between oral switch therapy and mortality.

## CONCLUSIONS

This study, representing one of the largest North American cohorts to date, adds to the growing body of evidence supporting the safety and efficacy of partial oral antibiotic therapy for select patients with IE. In carefully chosen cases, particularly those evaluated by a multidisciplinary endocarditis team, and including patients with injection drug use, oral stepdown therapy appears to be a viable option, consistent with European guidelines [7]. Patients with MRSA IE may also be considered for oral transition following thorough assessment by clinicians with expertise in cardiovascular infections.

## Supplementary Material

ofaf625_Supplementary_Data

## Supplementary Data

Supplementary materials are available at *Open Forum Infectious Diseases* online. Consisting of data provided by the authors to benefit the reader, the posted materials are not copyedited and are the sole responsibility of the authors, so questions or comments should be addressed to the corresponding author.

## Notes

***Author contributions.*** S. E.: Conceptualization, Methodology, Formal analysis, Investigation, Data curation, Writing – original draft, Writing – review & editing, Visualization, Supervision. B. C.: Methodology, Data curation, Formal analysis Writing – review & editing. T. K.: Methodology, Data curation, Formal analysis, Writing – review & editing. E. H.: Data curation, Writing – review & editing. T. A.: Writing – review & editing. C. C.: Data curation, Writing – review & editing. M. J.: Data curation, Writing – review & editing. J. G.: Writing – review & editing. L. S.: Writing – review & editing. C. A.: Writing – review & editing. V. W.: Writing – review & editing. H. R.: Writing – review & editing. M. S.: Writing – review & editing. T. L.: Writing – review & editing. K. K.: Writing – review & editing. A. M.: Writing – review & editing. D. O.: Writing – review & editing. G. L.: Writing – review & editing. A. T.: Writing – review & editing. A. L.: Writing – review & editing. D. G: Writing – review & editing. N. J. V. S.: Writing – review & editing. A. P.: Writing – review & editing. W. H.: Writing – review & editing. B. J. S.: Conceptualization, Resources, Data curation, Writing – review & editing, Visualization, Supervision.

***Ethics approval and patient consent.*** The study was approved by the University of Kentucky IRB (#71514). Patient consent was not required by the IRB.
