# Supplementary material for: Partial Oral Versus Intravenous Antibiotic Therapy for Endocarditis With Management by a Multidisciplinary Team: A Retrospective Cohort Study
Source: Open Forum Infect Dis. 2025 Oct 28;12(10):ofaf625. doi: 10.1093/ofid/ofaf625 (PMC12560757; doi:10.1093/ofid/ofaf625)
Supplement: ofaf625_Supplementary_Data [file ofaf625_supplementary_data.docx]

Supplemental Table 1. Detailed microbiologic data for all patients included in the study.

| **Organism** | **Entire Cohort**  **N = 236** |
| --- | --- |
| ***Methicillin-Resistant Staphylococcus Aureus, % (n)*** | 30.5 (72) |
| ***Methicillin-Susceptible Staphylococcus Aureus, % (n)*** | 28.8 (68) |
| ***Streptococcal Species, % (n)*** | 16.5 (39) |
| *Viridans Streptococcus species, % (n)* | 10.2 (24) |
| *Streptococcus pneumoniae, % (n)* | 1.3 (3) |
| *Group A Streptococcus pyogenes, % (n)* | 0.4 (1) |
| *Group B Streptococcus agalactiae, % (n)* | 2.5 (6) |
| *Group C Streptococcus dysgalactiae, % (n)* | 0.4 (1) |
| *Group G Streptococcus canis, % (n)* | 0.4 (1) |
| *Streptococcus bovis/gallolyticus species, % (n)* | 0.8 (2) |
| *Granulicatella adjacens, % (n)* | 0.4 (1) |
| ***Enterococcus species, % (n)*** | 13.6 (32) |
| *Enterococcus faecalis, % (n)* | 12.7 (30) |
| *Enterococcus faecium, % (n)* | 0.8 (2) |
| ***Coagulase Negative Staphylococcus, % (n)*** | 3.4 (8) |
| *Staphylococcus epidermidis, % (n)* | 1.7 (4) |
| *Staphylococcus lugdunensis, % (n)* | 0.8 (2) |
| *Staphylococcus hominis, % (n)* | 0.8 (2) |
| ***Serratia marcescens, % (n)*** | 3.8 (9) |
| ***Candida species, % (n)*** | 0.8 (2) |
| *Candida parapsiolosis, % (n)* | 0.4 (1) |
| *Candida glabrata, % (n)* | 0.4 (1) |
| ***HACEK Organisms, % (n)*** | 0.8 (2) |
| *Cardiobacterium hominis, % (n)* | 0.4 (1) |
| *Aggregatibacter aphorphilus, % (n)* | 0.4 (1) |
| ***Pseudomonas species, % (n)*** | 1.3 (3) |
| *Pseudomonas aeruginosa, % (n)* | 0.8 (2) |
| *Pseudomonas putida, % (n)* | 0.4 (1) |
| ***Rothia mucilaginosa, % (n)*** | 0.4 (1) |

Supplemental Table 2. Antimicrobial therapy and clinical outcomes for patients with methicillin- resistant *Staphylococcus aureus* infections treated with intravenous and oral antibiotics.

| ***Methicillin-Resistant Staphylococcus Aureus*** | | | |
| --- | --- | --- | --- |
| **Variable** | **Intravenous Only Therapy**  **N = 48** | **Intravenous with Oral Course Completion Therapy**  **N = 24** | **P Value** |
| **Intravenous Antimicrobial Therapy, % (n)** | 100 (48) | 100 (24) |  |
| Daptomycin, % (n) | 54.2 (26) | 83.3 (20) | 0.01 |
| Vancomycin, % (n) | 66.7 (32) | 45.8 (11) | 0.09 |
| Linezolid, % (n) | 2.1 (1) | 0 (0) | 0.48 |
| Ceftaroline, % (n) | 2.1 (1) | 8.3 (2) | 0.22 |
| Dalbavancin, % (n) | 2.1 (1) | 8.3 (2) | 0.22 |
|  |  |  |  |
| **Oral Course Completion Therapy, % (n)** | N/A | 24 (100) |  |
| **Days of Oral Course Completion Therapy, Median (IQR)** | N/A | 14 (9.3 - 17.5) |  |
| Linezolid, % (n) | N/A | 24 (100) |  |
|  |  |  |  |
| Total Duration of Intravenous with/- Oral Course Completion Therapy, Median (IQR) | 41 (18.8 - 42) | 41.5 (35.3 - 43) | 0.98 |
|  |  |  |  |
| **Oral Consolidation/Suppressive Therapy, % (n)** | 25.0 (12) | 20.8 (5) | 0.69 |
| Doxycycline, % (n) | 20.8 (10) | 16.7 (4) | 0.68 |
| Minocycline, % (n) | 2.1 (1) | 0 (0) | 0.48 |
| Trimethoprim/Sulfamethoxazole, % (n) | 2.1 (1) | 4.2 (1) | 0.61 |
|  |  |  |  |
| 30-Day Relapsed Infection, % (n) | 2.1 (1) | 0 (0) | 0.48 |
| 30-Day All Cause Readmission, % (n) | 25.0 (12) | 20.8 (5) | 0.69 |
| 30-Day Mortality, % (n) | 0 (0) | 0 (0) |  |
| 90-Day Relapsed Infection, % (n) | 2.1 (1) | 8.3 (2) | 0.22 |
| 90-Day All-Cause Readmission, % (n) | 31.3 (15) | 33.3 (8) | 0.86 |
| 90-Day Mortality, % (n) | 0 (0) | 0 (0) |  |

Supplemental Table 3. Antimicrobial therapy and clinical outcomes for patients with methicillin-susceptible *Staphylococcus aureus* infections treated with intravenous and oral antibiotics.

| ***Methicillin-Susceptible Staphylococcus Aureus*** | | | |
| --- | --- | --- | --- |
| **Variable** | **Intravenous Only Therapy**  **N = 42** | **Intravenous with Oral Course Completion Therapy**  **N = 26** | **P Value** |
| **Intravenous Antimicrobial Therapy, % (n)** | 100 (42) | 26 (100) |  |
| Cefazolin, % (n) | 83.3 (35) | 80.8 (21) | 0.79 |
| Nafcillin, % (n) | 23.8 (10) | 42.3 (11) | 0.11 |
| Daptomycin, % (n) | 9.5 (4) | 3.8 (1) | 0.38 |
| Cefepime, % (n) | 2.4 (1) | 0 (0) | 0.43 |
| Dalbavancin, % (n) | 7.1 (3) | 0 (0) | 0.17 |
|  |  |  |  |
| **Oral Therapy, % (n)** | N/A | 26 (100) |  |
| **Days of Oral Therapy, Median (IQR)** | N/A | 13 (8.8 - 14.8) |  |
| Linezolid with Cefadroxil, % (n) | N/A | 53.8 (14) |  |
| Doxycycline with Cefadroxil, % (n) | N/A | 3.8 (1) |  |
| Linezolid with Rifampin, % (n) | N/A | 3.8 (1) |  |
| Dicloxacillin with Rifampin, % (n) | N/A | 3.8 (1) |  |
| Levofloxacin with Cefadroxil, % (n) | N/A | 3.8 (1) |  |
| Cefadroxil, % (n) | N/A | 7.7 (2) |  |
| Linezolid, % (n) | N/A | 23.1 (6) |  |
|  |  |  |  |
| Total Duration of Intravenous/Oral Therapy, Median (IQR) | 40.5 (11.3 - 41) | 39.5 (28 - 42) | 0.09 |
|  |  |  |  |
| **Oral Consolidation/Suppressive Therapy, % (n)** | 33.3 (14) | 15.4 (4) | 0.11 |
| Cephalexin, % (n) | 14.3 (6) | 11.5 (3) | 0.74 |
| Cefadroxil, % (n) | 14.3 (6) | 0 (0) | 0.05 |
| Doxycycline, % (n) | 4.8 (2) | 0 (0) | 0.26 |
| Minocycline, % (n) | 0 (0) | 3.8 (1) | 0.21 |
|  |  |  |  |
| 30-Day Relapsed Infection, % (n) | 0 (0) | 0 (0) |  |
| 30-Day All Cause Readmission, % (n) | 35.7 (15) | 26.9 (7) | 0.45 |
| 30-Day Mortality, % (n) | 0 (0) | 0 (0) |  |
| 90-Day Relapsed Infection, % (n) | 0 (0) | 0 (0) |  |
| 90-Day All-Cause Readmission, % (n) | 40.5 (17) | 30.8 (8) | 0.42 |
| 90-Day Mortality, % (n) | 2.4 (1) | 7.7 (2) | 0.31 |

Supplemental Table 4. Antimicrobial therapy and clinical outcomes for patients with streptococcal infections treated with intravenous and oral antibiotics.

| ***Streptococcus* species** | | | |
| --- | --- | --- | --- |
| **Variable** | **Intravenous Only Therapy**  **N = 22** | **Intravenous with Oral Course Completion Therapy**  **N = 17** | **P Value** |
| **Intravenous Antimicrobial Therapy, % (n)** | 100 (22) | 100 (17) |  |
| Ceftriaxone, % (n) | 95.5 (21) | 94.1 (16) | 0.85 |
| Penicillin G, % (n) | 4.5 (1) | 5.9 (1) | 0.85 |
| Vancomycin, % (n) | 4.5 (1) | 5.9 (1) | 0.85 |
| Cefepime, % (n) | 4.5 (1) | 0 (0) | 0.38 |
| Cefazolin, % (n) | 4.5 (1) | 0 (0) | 0.38 |
| Daptomycin, % (n) | 0 (0) | 5.9 (1) | 0.25 |
|  |  |  |  |
| **Oral Therapy, % (n)** | N/A | 100 (17) |  |
| **Days of Oral Therapy, Median (IQR)** | N/A | 16 (10 - 24) |  |
| Linezolid with Amoxicillin, % (n) | N/A | 17.6 (3) |  |
| Levofloxacin with Amoxicillin, % (nn | N/A | 23.5 (4) |  |
| Linezolid with Levofloxacin, % (n) | N/A | 41.2 (7) |  |
| Linezolid, % (n) | N/A | 17.6 (3) |  |
|  |  |  |  |
| Total Duration of Intravenous/Oral Therapy, Median (IQR) | 41 (27 - 41) | 41 (28 - 42) | 0.49 |
|  |  |  |  |
| **Oral Consolidation/Suppressive Therapy, % (n)** | 18.2 (4) | 11.8 (2) | 0.59 |
| Amoxicillin, % (n) | 9.1 (2) | 5.9 (1) | 0.71 |
| Cefuroxime, % (n) | 4.5 (1) | 5.9 (1) | 0.85 |
| Cefdinir, % (n) | 4.5 (1) | 0 (0) | 0.38 |
|  |  |  |  |
| 30-Day Relapsed Infection, % (n) | 0 (0) | 0 (0) |  |
| 30-Day All Cause Readmission, % (n) | 27.3 (6) | 29.4 (5) | 0.89 |
| 30-Day Mortality, % (n) | 0 (0) | 0 (0) |  |
| 90-Day Relapsed Infection, % (n) | 0 (0) | 0 (0) |  |
| 90-Day All-Cause Readmission, % (n) | 27.3 (6) | 41.2 (7) | 0.37 |
| 90-Day Mortality, % (n) | 0 (0) | 17.6 (3) | 0.05 |

Supplemental Table 5. Antimicrobial therapy and clinical outcomes for patients with *Enterococcus* infections treated with intravenous and oral antibiotics.

| ***Enterococcus* species** | | | |
| --- | --- | --- | --- |
| **Variable** | **Intravenous Only Therapy**  **N = 12** | **Intravenous with Oral Course Completion Therapy**  **N = 20** | **P Value** |
| **Intravenous Therapy, % (n)** | 100 (12) | 100 (20) |  |
| Ampicillin with Ceftriaxone, % (n) | 100 (12) | 100 (20) |  |
| Daptomycin, % (n) | 16.7 (2) | 15.0 (3) | 0.90 |
| Vancomycin, % (n) | 8.3 (1) | 5.0 (1) | 0.71 |
|  |  |  |  |
| **Oral Therapy, % (n)** | N/A | 100 (20) |  |
| **Days of Oral Course Therapy, Median (IQR)** | N/A | 20 (13.8 - 28.3) |  |
| Linezolid with Amoxicillin, % (n) | N/A | 45.0 (9) |  |
| Levofloxacin with Amoxicillin, % (n) | N/A | 5.0 (1) |  |
| Linezolid with Levofloxacin, % (n) | N/A | 15.0 (3) |  |
| Moxifloxacin with Amoxicillin, % (n) | N/A | 25.0 (5) |  |
| Linezolid, % (n) | N/A | 5.0 (1) |  |
| Amoxicillin, % (n) | N/A | 5.0 (1) |  |
|  |  |  |  |
| Total Duration of Intravenous/Oral Course Therapy, Median (IQR) | 41 (37.3 - 42) | 41 (40.8 - 42) | 0.20 |
|  |  |  |  |
| **Oral Consolidation/Suppressive Therapy, % (n)** | 33.3 (4) | 15.0 (3) | 0.23 |
| Amoxicillin, % (n) | 33.3 (4) | 15.0 (3) | 0.23 |
|  |  |  |  |
| 30-Day Relapsed Infection, % (n) | 0 (0) | 5.0 (1) | 0.44 |
| 30-Day All Cause Readmission, % (n) | 33.3 (4) | 20.0 (4) | 0.41 |
| 30-Day Mortality, % (n) | 8.3 (1) | 0 (0) | 0.20 |
| 90-Day Relapsed Infection, % (n) | 0 (0) | 5.0 (1) | 0.44 |
| 90-Day All-Cause Readmission, % (n) | 33.3 (4) | 35.0 (7) | 0.92 |
| 90-Day Mortality, % (n) | 8.3 (1) | 5.0 (1) | 0.71 |

Supplemental Table 6. Demographic data for patients with isolated right sided endocarditis treated with intravenous and partial oral therapy.

| **Variable** | **Intravenous Only**  **N = 50** | **Oral Transition**  **N = 37** | **P Value** |
| --- | --- | --- | --- |
| Age, Median (IQR) | 35 (31 - 40.8) | 35 (29 - 40) | 0.64 |
| Male, % (n) | 52.0 (26) | 48.6 (18) | 0.76 |
| Female, % (n) | 48.0 (24) | 51.4 (19) |  |
| White, % (n) | 90.0 (45) | 97.3 (36) | 0.10 |
| Black, % (n) | 8.0 (4) | 0 (0) |  |
| Hispanic, % (n) | 0 (0) | 2.8 (1) |  |
| Other, % (n) | 2.0 (1) | 0 (0) |  |
| Injection Drug Use, % (n) | 88.0 (44) | 83.8 (31) | 0.58 |
| Hepatitis C Viremia, % (n) | 60.0 (30) | 35.1 (13) | 0.02 |
| Previous Infective Endocarditis Admission, % (n) | 46.0 (23) | 45.9 (17) | 0.99 |
| Dental Disease, % (n) | 38.0 (19) | 21.6 (8) | 0.10 |
| Diabetes Mellitus, % (n) | 6.0 (3) | 8.1 (3) | 0.70 |
| Chronic Dialysis, % (n) | 2.0 (1) | 0 (0) | 0.39 |
| Outside Hospital Transfer, % (n) | 36.0 (18) | 48.6 (18) | 0.24 |

Supplemental Table 7. Microbiologic data for patients with isolated right sided endocarditis treated with intravenous and partial oral therapy.

| **Variable** | **Intravenous Only**  **N = 50** | **Oral Transition**  **N = 37** | **P Value** |
| --- | --- | --- | --- |
| *Methicillin-Resistant Staphylococcus aureus, % (n)* | 46.0 (23) | 48.6 (18) | 0.81 |
| *Methicillin-Susceptible Staphylococcus aureus, % (n)* | 40.0 (20) | 37.8 (14) | 0.84 |
| *Enterococcus faecalis, % (n)* | 2.0 (1) | 2.7 (1) | 0.83 |
| *Streptococcal species, % (n)* | 8.0 (4) | 10.8 (4) | 0.66 |
| *Serratia marcescens, % (n)* | 4.0 (2) | 0 (0) | 0.22 |

Supplemental Table 8. Outcomes data for patients with isolated right sided endocarditis treated with intravenous and partial oral therapy.

| **Variable** | **Intravenous Only**  **N = 50** | **Oral Transition**  **N = 37** | **P Value** |
| --- | --- | --- | --- |
| Percutaneous Mechanical Aspiration, % (n) | 8.0 (4) | 13.5 (5) | 0.41 |
| Patient Directed Discharge, % (n) | 30.0 (15) | 27.0 (10) | 0.72 |
| Length of Stay, Median (IQR) | 28 (12.3 - 46) | 26 (15 - 36) | 0.23 |
| 30-Day Relapsed Infection, % (n) | 0 (0) | 0 (0) | N/A |
| 30-Day Readmission, % (n) | 36.0 (18) | 32.4 (12) | 0.73 |
| 30-Day All-Cause Mortality, % (n) | 0 (0) | 0 (0) | N/A |
| 90-Day Relapsed Infection, % (n) | 0 (0) | 5.4 (2) | 0.10 |
| 90-Day Readmission, % (n) | 40.0 (20) | 37.8 (14) | 0.84 |
| 90-Day All-Cause Mortality, % (n) | 0 (0) | 2.7 (1) | 0.25 |
| Composite of 90-Day All-Cause Mortality and Relapsed Infection, % (n) | 0 (0) | 8.1 (3) | 0.04 |

Supplemental Table 9. Follow-up rates for patients with isolated right sided endocarditis treated with intravenous and partial oral therapy.

| **Variable** | **Intravenous Only**  **N = 50** | **Oral Transition**  **N = 37** | **P Value** |
| --- | --- | --- | --- |
| Outpatient University of Kentucky Follow-up | 68.0 (34) | 75.7 (28) | 0.44 |
| Outpatient University of Kentucky Infectious Disease Follow-up, % (n) | 59.0 (29) | 75.7 (28) | 0.11 |
| Outpatient University of Kentucky Cardiac Surgery Follow-up, % (n) | 56.0 (28) | 35.1 (13) | 0.05 |
| Outpatient University of Kentucky Cardiology Follow-up, % (n) | 40.0 (20) | 16.2 (6) | 0.02 |
| Outpatient University of Kentucky Neurosurgery Follow-up, % (n) | 4.0 (2) | 5.4 (2) | 0.76 |
| Outpatient University of Kentucky Neurology Follow-up, % (n) | 2.0 (1) | 2.7 (1) | 0.83 |
| Outpatient University of Kentucky Addiction Medicine Follow-up, % (n) | 16.0 (8) | 5.4 (2) | 0.13 |
